# Physical activity level one year following admission to the intensive care unit for COVID-19

**DOI:** 10.1038/s41598-025-96775-0

**Published:** 2025-04-22

**Authors:** Netha Hussain, Carina M. Samuelsson, Mats Börjesson, Carina U. Persson

**Affiliations:** 1 Department of Radiology, Sahlgrenska University Hospital, Region Västra Götaland, Gothenburg, Sweden; 2 Department of Occupational Therapy and Physiotherapy, Sahlgrenska University Hospital/Östra, Region Västra Götaland, Gothenburg, Sweden; 3 Center of Lifestyle Intervention, Institute of Medicine, University of Gothenburg, Sahlgrenska Academy and Sahlgrenska University hospital, Region Västra Götaland, Gothenburg, Sweden; 4 Department of Clinical Neuroscience, Rehabilitation Medicine, Institute of Neuroscience and Physiology, Sahlgrenska Academy, University of Gothenburg, Gothenburg, Sweden

**Keywords:** Diseases, Medical research

## Abstract

While regular physical activity is associated to multiple health effects, a COVID-19 infection may affect the ability to be physically active, due to muscular, cardiac and pulmonary complications. Therefore, the aim of this study was to describe the physical activity level and to identify factors at baseline that are associated with being physically inactive at one year following intensive care unit (ICU) admission for COVID-19. The dependent variable, being physically inactive, was assessed using the Saltin-Grimby Physical Activity Level Scale and the International Physical Activity Questionnaire Short Form (IPAQ-SF). To identify any predictors, univariable and multivariable logistic regression analyses were performed. A total of 105 (57.7%) and 95 (52.2%) survivors responded to the two scales. Being physically inactive was self-reported by 25.7% and 22.1%, respectively. Using the Saltin-Grimby Physical Activity Level Scale, longer stay at the ICU [odds ratio 1.05 (95% confidence interval (95% CI) 1.02–1.08)] and diabetes mellitus [3.92 (1.30-11.55)] were identified as predictors, while using the IPAQ-SF, longer stay at the ICU [odds ratio 1.04 (95% CI 1.01–1.07)] was the predictor for being physically inactive at one year following COVID-19.

**Supplementary Information:**

The online version contains supplementary material available at 10.1038/s41598-025-96775-0.

## Introduction

Physical inactivity is a significant contributor to cardiovascular diseases, diabetes and obesity^1^ and the fourth leading cause of death worldwide^2^. Globally, self-reported data have shown that 27% of the adults do not meet the levels of physical activity recommended by the World Health Organization (WHO)^3^. In Sweden, previous studies indicate that 19% of the adults self-report as physically inactive^4^, although these figures may underestimate the true prevalence when compared to objective assessment methods such as accelerometry^5^. A recent and large nationwide accelerometer-based study in Sweden revealed that individuals spent 55% of their waking time engaging in sedentary activities^6^. Given the high prevalence and wide-ranging health consequences of physical inactivity, it is a global priority to understand the underlying factors contributing to the same^7–9^.

The prevalence of long-term post-COVID physical inactivity of individuals who were admitted to the ICU for COVID-19 in Sweden has not been sufficiently investigated. Previous studies have shown that 34% of those admitted to the intensive care unit (ICU) after COVID-19 reported difficulty in performing their usual activities while 29% reported problems with walking at one-year follow-up^10^. Another study showed that the self-reported physical activity levels of individuals at one-year after ICU admission for COVID-19 did not reach pre-COVID levels^11^. Furthermore, 74% of ICU admitted individuals reported physical symptoms in a questionnaire survey^12^ and 68% reported reduced fitness in a telephonic interview^13^ at a one year follow-up after ICU admission for COVID-19. Therefore, ICU admission and COVID-19 are likely to cause long-term conditions that affect an individual’s ability to perform physical activity.

A few studies have examined the relationship between pre-COVID physical activity and severity or sequelae of COVID-19^14,15^. However, to date and to the best of our knowledge, there is currently no published literature related to early predictors of physical activity level one year following ICU admission for COVID-19. Higher age^16^, female sex^17^, co-morbidities at the time of ICU admission and complications during the ICU stay are generally associated with poorer long-term outcomes^18^. However, it remains unclear whether these factors also influence physical inactivity one year after COVID-19. Conversely, a previous study from the same cohort as the current study showed that younger age was a predictor of self-reported fatigue at one year follow-up after ICU admission for COVID-19^19^. Understanding the predictors of physical inactivity after severe COVID-19 is crucial for early prevention of sequelae, promoting physical activity and managing and supporting individuals at risk for physical inactivity.

To address these knowledge gaps, the aim of this study was to describe the physical activity level and to identify factors at baseline that are associated with being physically inactive at one year following ICU admission for COVID-19. Based on findings from previous research^16,17^ and clinical reasoning, our hypothesis was that self-reported physical inactivity at one year after ICU admission for COVID-19 among Swedish adults is associated with younger age, female sex, presence of comorbidities and markers of disease severity such as longer stay at the ICU and complications that arose during the ICU stay.

## Methods

### Study design

The participants of the study were obtained from the Gothenburg Recovery and Rehabilitation after COVID-19 and Intensive Care Unit (GOT-RECOV-19 ICU) cohort. GOT-RECOV-19 ICU is a multicenter study, with four prior publications^19–22^. The study was officially registered at “FoU i Sverige” (researchweb.org) on May 28, 2020 (ID number: 274477). It followed a cross-sectional, retrospective, and longitudinal design approach.

### Inclusion and exclusion criteria

**Fig. 1:**
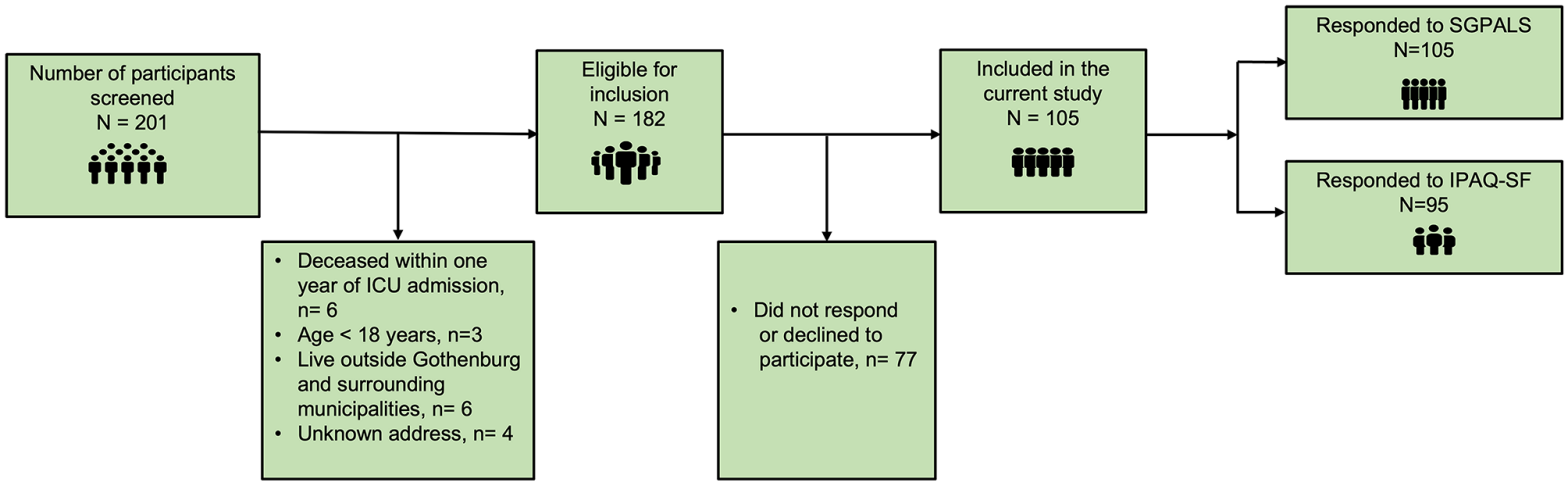
Flowchart of the inclusion process.

A request for study participation was sent to all adult individuals (defined as 18 years or above) who received treatment for COVID-19 in any of the five Intensive Care Units (ICUs) at the Sahlgrenska University Hospital/Östra between 1 March 2020 and 30 June 2020, who were alive one year after ICU admission, and had the code ICD-10-SE, UO7.1 (i.e., COVID-19 virus detected) as the main diagnosis. Those not listed as Swedish residents in the Swedish Population Register or not residing in Gothenburg or adjacent municipalities were not considered for the study. The selection process is illustrated in Fig. 1.

### Procedures

In March 2021, we collected the medical record data of individuals admitted to the ICU with a primary diagnosis of COVID-19 from the Sahlgrenska University Hospital’s Output Unit. Out of the 259 potential participants who were identified, 58 had passed away before the one-year follow-up. After exclusion of 19 people who did not meet the selection criteria, 182 individuals were invited to participate in the study (Fig. 1). If there was no response to the initial invitation, one or two reminders were sent out. In the second reminder, the potential participants were given an option to respond solely to the questionnaires and send them back postally or answer digitally using a link.

Informed written consent was obtained at the time of onsite clinical evaluations. Those who did not attend the onsite clinical evaluations but completed the questionnaires at home and returned them to us postally or digitally were considered to have given informed consent. Ethical approval was granted by the Swedish Ethical Review Authority on September 24, 2020 (ID number: 2020–03264), which also approved the sending of the reminder mails to the participants on June 29, 2021 (ID number: 2021–03220). All authors have access to the identification key to the pseudo-anonymized participants’ data. All methods were performed in accordance with the relevant guidelines and regulations.

### Assessment of independent variables at baseline

For those who agreed to participate, baseline information was extracted from the medical records. This information included age, sex, length of ICU stay and presence of comorbidities (hypertension; diabetes mellitus; chronic kidney disease; coronary heart disease; chronic heart failure; chronic obstructive pulmonary disease and asthma). Any complications during the care period in the ICU such as sepsis, acute respiratory distress syndrome (ARDS) and embolic events (myocardial infarction, ischemic stroke, pulmonary embolism, deep vein thrombosis) (yes/no) were also recorded. The criterion for multimorbidity was the presence of two or more concurrent chronic conditions^23^. The clinical assessments were carried out by two physiotherapist researchers (CMS, CUP) at the Sahlgrenska University Hospital/Östra.

### Primary outcomes

The primary outcome for this study was being physically inactive, defined as the category ‘physically inactive’ using the Saltin-Grimby Physical Activity Level Scale (SGPALS, https://www.gu.se/sites/default/files/2020-04/sgpals-english-gu-january-2016.pdf)^24,25^ and the category ‘inactive’ using the International Physical Activity Questionnaire – Short Form (IPAQ-SF, https://sites.google.com/view/ipaq/home)^26,27^, as described in detail below. The SGPALS assesses the physical activity level over the past year, whereas the IPAQ-SF assesses the physical activity level over the past week, allowing this study to assess both long-term and short-term physical inactivity. The data collection for the primary outcomes started in March 2021 and was performed consecutively at one year following the participants´ admission to the ICU.

The SGPALS is a one-item ordinal scale with four response categories^24,25^. A higher score indicates a higher average self-reported physical activity level during the past year. The scores from one to four were defined as: (1) Physically inactive, (2) Some light physical activity, (3) Regular physical activity and training, and (4) Regular hard physical training for competitive sports^25^. The concurrent validity of SGPALS against accelerometry data has been found to be weak to moderate^28^, its reproducibility to be acceptable^25,29,30^ and its predictive validity for cardiovascular risk factors to be high^31^.

The IPAQ-SF is a 4-item questionnaire containing self-reported physical activity performed during the last 7 days^26^. The IPAQ-SF assesses the time spent/engaged in moderate and vigorous physical activity, as well as in walking and sitting. The IPAQ-SF score was then classified as: (1) Inactive, (2) Minimally active and (3) Health enhancing physical activity (HEPA), according to the IPAQ-SF scoring protocol^26^ based on metabolic equivalents (METs) minutes^27^. In the present study, the Swedish language version of the IPAQ-SF was administered onsite at the Sahlgrenska University Hospital/Östra or sent home/digitally. The IPAQ-SF has been found to have moderate criterion validity with reference to accelerometry data in Swedish adults^32^. IPAQ-SF is known to overestimate physical activity level in comparison with accelerometry data^32,33^. The IPAQ-SF has previously been used in healthy individuals for evaluating physical activity during the COVID-19 lockdown^34,35^ as well as in ICU-admitted individuals one year after COVID-19^11^. For the participants who performed the assessments onsite, the SGPALS and the IPAQ-SF were performed as seventh and eighth in a battery of twelve tests. The printed questionnaires were provided to the participants, who subsequently filled them out. Those who received postal or digital questionnaires were free to answer them in any order. Both questionnaires are not protected by copyright and hence no permission to use them was required.

We collaborated with the Swedish COVID association (https://covidforeningen.se/, a registered body that aims to increase awareness about COVID-19 and its sequelae). The members of the association prioritized the symptoms and sequelae being studied as part of the GOT-RECOV-19 ICU. They ranked physical inactivity as the third most critical sequela needing research.

### Statistical analyses

The statistical analyses were performed using the IBM Statistical Package for Social Sciences (SPSS) software, version 28. The figures were prepared using Microsoft Office 365 PowerPoint (2021) and Adobe Photoshop v20. The power calculation for this cohort has been elaborated in a previous publication from the same study cohort, where the lowest prevalence of post-COVID-19 fatigue was taken as 40%^36^, compared to 20%^37^ in the general population. The expected drop-out rate was taken as 40%, required power was 80% and a margin of error was 0.05 (two tailed), for which the required lowest sample size was 105^19^. A p-value less than 0.05 (two-tailed) was designated as the threshold for statistical significance. To check for significant differences between the participants (*n* = 105) and non-participants (*n* = 77), Mann-Whitney U test was used for age and length of ICU stay, while Chi square test was used for sex. Descriptive statistics, such as means and standard deviations (SD) or medians and interquartile ranges (IQR) were reported for continuous data, medians and IQRs for ordinal data and n (%) for nominal data.

Univariable and multivariable binary logistic regression analyses were planned to be performed to identify any predictors for being physical inactive at one year after ICU admission due to COVID-19. When the dependent variable was based on SGPALS scores, the categories were dichotomized into ‘Physically inactive’ (score 1) and ‘Physically active’ (scores 2–4). When the dependent variable was based on IPAQ-SF scores, the ordinal categories were dichotomized into ‘Physically inactive’ (category ‘Inactive’) and ‘Physically active’ (categories ‘Minimally active’ and ‘HEPA’). For simplicity and ease of understanding, we chose to dichotomize SGPALS and IPAQ-SF into the same variables, although these scales assess physical inactivity at different time points and represent slightly different constructs.

The independent variables were, age and length of stay at the ICU (as covariates) and sex, hypertension, diabetes mellitus, coronary heart disease, need for invasive ventilation and sepsis (as factors). Independent variables with p-values < 0.1 in the univariable logistic regression, as well as the demographic factors of age and sex were included in the multivariable logistic regression models. Any multicollinearity between the independent variables was checked using the Spearman’s rank correlation coefficient, where correlation coefficients of ≥ 0.7 were deemed as multicollinear^38^.

The result of the regression analyses was presented as odds ratios (OR) with their 95% confidence intervals (CI) and p-values. Goodness-of-fit was tested using the Hosmer-Lemeshow test. Cox & Snell and Nagelkerke pseudo R^2^ were used to assess the percentage of improvement in fit. The area under the Receiver Operating Curve (ROC) was calculated for each of the final prediction models, with 70–79% considered as acceptable, 80–89% as excellent and 90–100% as outstanding predictive accuracy^39^. Lastly, a post-hoc analysis using Mann-Whitney U test was performed to find if any significant differences existed between females and males in terms of length of stay at the ICU.

## Results

Out of the 182 COVID-19 survivors who met the inclusion criteria, 105 (57.7%) completed the SGPALS and 95 (52.2%) responded to the IPAQ-SF. Of those who completed the SGPALS, 78 participants did so during their visit to the hospital, 26 responded postally and one responded digitally. Ten participants, three who completed the SGPALS on-site and seven who sent in their responses postally, did not fill out the IPAQ-SF. No statistically significant differences in terms of age, sex, or length of stay at the ICU were found between the 105 participants and the 77 individuals who declined to participate or did not respond to the study request.

The demographic characteristics, comorbidities, complications during the ICU and other baseline characteristics of the study group have already been presented in a previous study^19^. No multicollinearity was found between the dependent variables. Dichotomized data for physically active and inactive individuals according to both SGPALS and IPAQ-SF are shown in Table 1. Hypertension was the most common comorbidity, and more than one-thirds of the participants were multimorbid. Out of the participants with diabetes mellitus, one had type I diabetes mellitus (T1DM), the remaining participants had type II diabetes mellitus (T2DM). Over four in five participants required mechanical ventilation during their ICU stay.

**Table 1:** Demographic characteristics of the participants at the time of admission to intensive care unit due to severe COVID-19.

Characteristics (*n* = 105)	All participants [Mean ± SD, median (IQR), *n* (%)]			
	SGPALS (*n* = 105)		IPAQ-SF (*n* = 95)	
	Physically active (*n* = 78)	Physically inactive (*n* = 27)	Physically active (*n* = 74)	Physically inactive (*n* = 21)
Age, years	57.89 ± 11.68	59.01 ± 12.44	58.29 ± 11.38	55.41 ± 14.26
Female	19 (24.4)	6 (22.2)	19 (25.7)	3 (14.3)
Male	59 (75.6)	21 (77.8)	55 (74.3)	18 (85.7)
BMI, kg/m^2^, (*n* = 54)	28.95 ± 7.61 (*n* = 40)	31.87 ± 8.41 (*n* = 15)	29.22 ± 7.98 (*n* = 42)	31.49 ± 9.11 (*n* = 8)
Length of stay in ICU, days				
All participants	12 (6.75–20)	28 (12–37)	13 (7-22.25)	27 (11.5–37.5)
Female	7 (4–14)	8.5 (6.5-13.25)	7 (4–15)	9 (–)
Male	14 (8–23)	33 (24.5–38)	16 (8–24)	28.5 (15–49)
Type of respiratory support				
Mask or nasal cannula	3 (3.8)	1 (3.7)	3 (4.1)	1 (4.8)
High flow oxygen therapy	14 (17.9)	1 (3.7)	12 (16.2)	2 (9.5)
Mechanical ventilation	61 (78.2)	3 (92.6)	59 (79.7)	18 (85.7)
Comorbidities				
Multimorbidity*	26 (33.33)	16 (59.26)	25 (33.78)	10 (47.61)
Hypertension	29 (37.2)	15 (55.6)	26 (35.1)	10 (47.6)
Coronary heart disease	19 (24.4)	8 (29.6)	21 (28.4)	4 (19.0)
Diabetes mellitus	13 (16.7)	10 (37.0)	14 (18.9)	6 (28.6)
Asthma	6 (7.7)	2 (7.4)	6 (8.1)	1 (4.8)
Chronic kidney disease	5 (6.4)	1 (3.7)	4 (5.4)	0 (0%)
Chronic heart failure	1 (1.3)	3 (11.1)	3 (4.1)	1 (4.8)
Chronic obstructive pulmonary disease	2 (2.6)	0 (0%)	2 (2.7)	0 (0%)
Complications during ICU admission				
ARDS	39 (50.0)	17 (63.0)	40 (54.1)	13 (61.9)
Sepsis	40 (51.3)	15 (55.6)	38 (51.4)	13 (61.9)
Embolic event	5 (6.4)	1 (3.7)	4 (5.4)	1 (4.8)

*SD* standard deviation, *IQR* interquartile range, *BMI* body mass index, *ARDS* acute respiratory distress syndrome. *Multimorbidity refers to the co-occurrence of two or more comorbidities.

### SGPALS

**Fig. 2:**
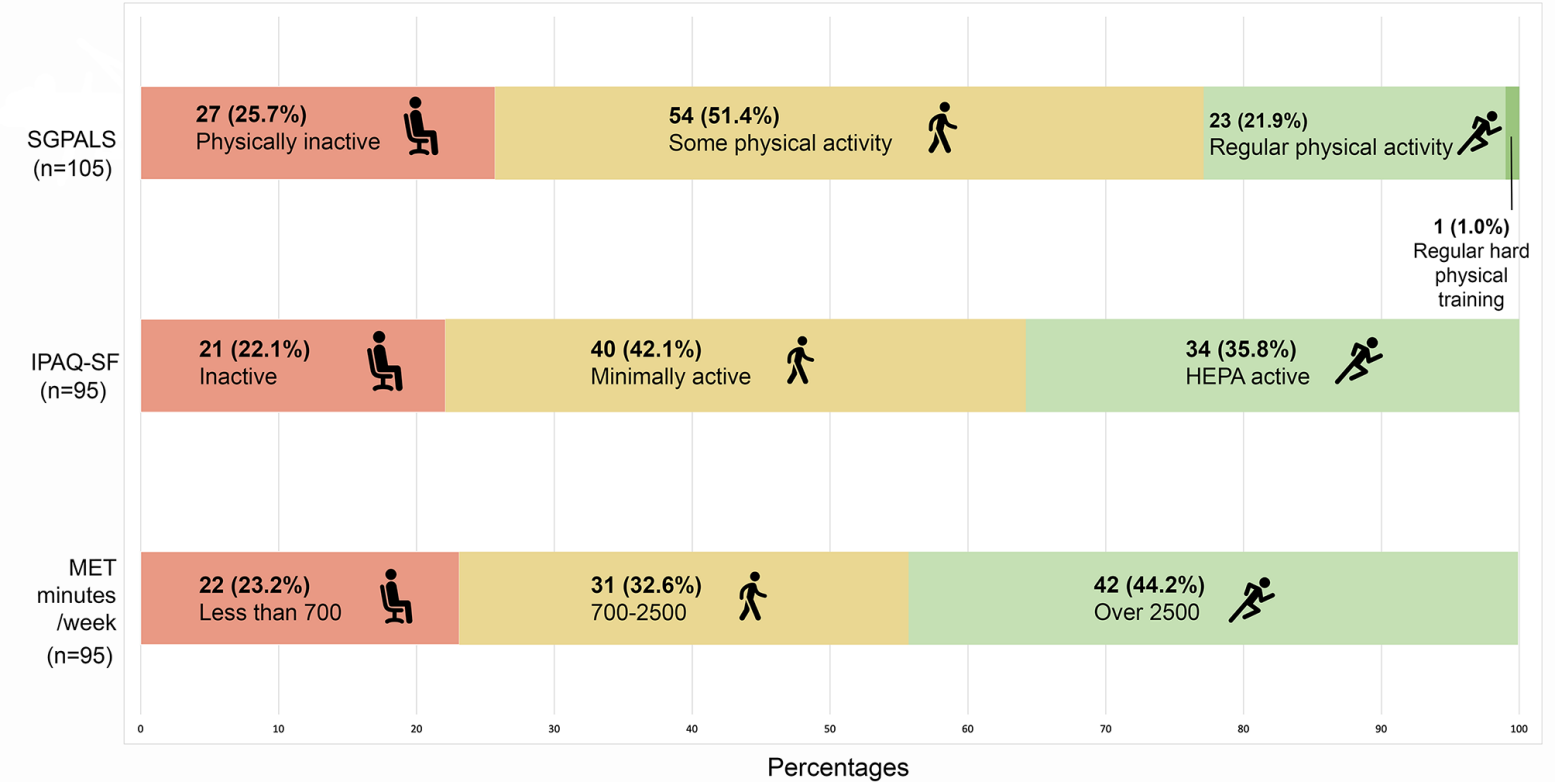
The proportions of participants with various physical activity levels as assessed using the SGPALS, the IPAQ-SF and MET minutes/week. *SGPALS* the Saltin-Grimby physical activity scale, *IPAQ-SF* the international physical activity questionnaire short form, *MET* metabolic equivalents, *HEPA* Health-enhancing physical activity.

Roughly, a quarter of the participants reported being physically inactive according to SGPALS. Approximately half of the participants reported being physically active to some extent, while only one in four reported being engaged in ´regular´ or ´regular to hard´ physical training (Fig. 2). Using univariable logistic regression, longer stay at the ICU and diabetes mellitus were found to be significant predictors for being physically inactive (Table 2).

**Table 2:** Univariable logistic regression analyses presenting odds ratios with 95% confidence intervals and p-values for the prediction of physical inactivity in ICU-admitted individuals at one year after COVID-19.

Independent variable	Univariable logistic regression	
	Odds ratio (95% CI)	*P*-value
**Dependent variable: SGPALS**		
Age	1.01 (0.97–1.05)	0.67
Female sex (male sex = ref.)	0.89 (0.31–2.52)	0.82
Length of stay in ICU	**1.04 (1.02–1.07)**	**< 0.002**
Hypertension	2.11 (0.87–5.12)	0.10
Diabetes mellitus	**2.94 (1.10–7.85)**	**0.03**
Coronary heart disease	1.31 (0.49–3.47)	0.59
Need for invasive ventilation	3.23 (0.69–15.07)	0.14
Sepsis	1.19 (0.49–2.86)	0.70
**Dependent variable: IPAQ-SF**		
Age	0.98 (0.94–1.02)	0.33
Female sex (male sex = ref.)	0.48 (0.13–1.82)	0.28
Length of stay in ICU	**1.04 (1.01–1.06)**	**0.004**
Hypertension	1.68 (0.63–4.47)	0.30
Diabetes mellitus	1.71 (0.56–5.21)	0.34
Coronary heart disease	0.59 (0.17–1.97)	0.40
Need for invasive ventilation	1.40 (0.36–5.42)	0.63
Sepsis	1.54 (0.57–4.15)	0.40

*SGPALS* The Saltin Grimby physical activity level scale, *IPAQ-SF* international physical activity questionnaire short form, *ICU* intensive care unit. Statistically significant values are in bold.

**Fig. 3:**
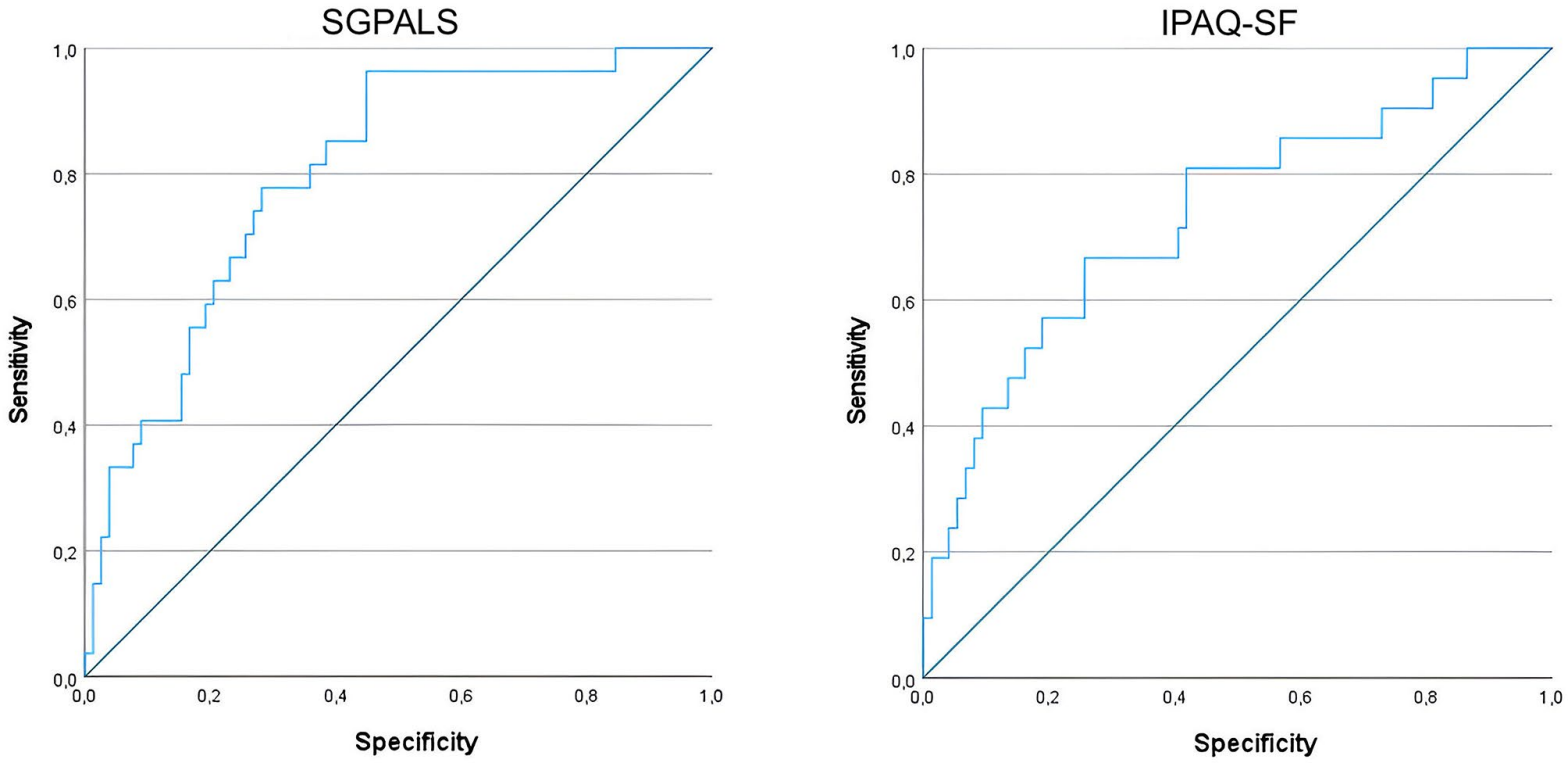
Receiver operating characteristic (ROC) curve for multivariable regression models with SGPALS (left) and IPAQ-SF (right) as dependent variables.

Multivariable logistic regression analysis identified length of stay at the ICU and diabetes mellitus as predictors for being physically inactive using SGPALS at one-year follow-up. The probability for being physically inactive was 5% higher per additional day spent in ICU. Similarly, odds ratio for being physically inactive is about four times higher for subjects with diabetes mellitus as compared with those that do not have diabetes mellitus at one-year following COVID-19 (Table 3). The predictive accuracy of this model was estimated to be 80.1% [CI: 70.9–89.2], interpreted as excellent accuracy. The ROC curves for the multivariable models with SGPALS and IPAQ-SF as dependent variables are shown in Fig. 3.

**Table 3:** Multivariable analyses presenting odds ratios with 95% confidence intervals and p-values for the prediction of physical inactivity in ICU-admitted individuals at one year after COVID-19 using the SGPALS and the IPAQ-SF as dependent variables.

Independent variable	Multivariable logistic regression	
	Odds ratio (95% CI)	*P*-value
**Dependent variable: SGPALS**		
Age	0.99 (0.94–1.03)	0.58
Female sex (male sex = ref.)	1.75 (0.52–5.82)	0.37
Length of stay in ICU	**1.05 (1.02–1.08)**	**0.001**
Diabetes mellitus	**3.92 (1.30-11.55)**	**0.01**
**Dependent variable: IPAQ-SF**		
Age	0.97 (0.92–1.01)	0.13
Female sex (male sex = ref.)	0.85 (0.21–3.51)	0.83
Length of stay in ICU	**1.04 (1.01–1.07)**	**0.004**

The significance of Hosmer-Lemeshow test was 0.30 and 0.92 for the models with SGPALS and IPAQ-SF as dependent variables respectively. The Cox and Snell R^2^ and Nagelkerke R^2^ were 0.18 & 0.26 for the SGPALS model, and 0.13 & 0.20 respectively for the IPAQ-SF model. *SGPALS* the Saltin Grimby physical activity level scale, *IPAQ-SF* international physical activity questionnaire short form, *ICU* intensive care unit. Statistically significant values are in bold.

### IPAQ-SF

Around a quarter of the participants reported being physically inactive according to IPAQ-SF. Two in five reported being minimally physically active, while only a third reported being physically active at health enhancing levels (Fig. 2). Univariable regression showed length of stay at the ICU to be a significant predictor for being inactive (Table 2). A multivariable model with age, sex and length of ICU stay showed that length of ICU stay was the only significant predictor [OR:1.04 (CI:1.01–1.07)], with the model showing acceptable 73.4% [CI: 60.7–86.1] accuracy (Table 3).

The distribution of physical activity levels of the cohort according to SGPALS, IPAQ-SF and MET minutes is shown in Fig. 2. The results of the univariable and multivariable logistic regression analyses are shown in Tables 2 and 3, respectively. Box plots derived from the univariable, and multivariable regression analyses are displayed in Figures S1 (also including comorbidities as independent variables) and S2 of the supplementary materials. No multicollinearity was found between the significant independent variables for the multivariable models. Age and sex were not found to be significant predictors of both SGPALS and IPAQ-SF. Post hoc analysis revealed statistically significant differences (*p* < 0.001) between length of stay at the ICU between males and females.

## Discussion

In this one-year follow-up, roughly a quarter of the participants reported to be physically inactive one year following ICU admission for COVID-19. To our knowledge, this is the first study that has identified predictors of being physically inactive at one year following admission to ICU due to COVID-19. Our hypothesis that increased length of stay at the ICU was a predictor of being physically inactive at one year follow-up according to IPAQ-SF (related to the past week) and SGPALS (related to the past year) was confirmed. Furthermore, based on the SGPALS, diabetes mellitus emerged as a risk factor for being physically inactive at one-year follow-up. However, our hypotheses that age, sex, hypertension, coronary heart disease, need for invasive ventilation, and sepsis predict physical inactivity at one year after ICU admission for COVID-19, could not be confirmed. More participants responded to the SGPALS than IPAQ-SF, possibly because SGPALS is shorter, it only consists of one single-item and takes less time to complete.

The proportions of physical inactivity were found to be 22.1–25.7% in the current study, slightly above the 19% rate in the general Swedish population, as assessed using IPAQ^4^. Similarly, physical inactivity as assessed using SGPALS was reported by 24.4% of the females and by 22.2% of the males in our study, which is greater than the 9% and the 12% reported in a population-based study from Sweden prior to the COVID-19 pandemic^31^. The physical inactivity level in our study is also higher than that of a pre-COVID population study with mainly healthcare workers in Sweden, were 15% of the respondents self-reported physical inactivity^40^. Furthermore, it is important to recognize that both the SGPALS and the IPAQ have shown to underestimate self-reported physical inactivity levels in relation to objective assessments such as accelerometry^28,33^, in which case the actual level of physical inactivity is likely considerably higher than what our study reveals, indicating a scope for lifestyle improvement in individuals after ICU admission for COVID-19. These results may also be affected by fatigue, which has been reported by 64% of the study cohort^19^ as well as post-intensive care syndrome (PICS)^41^.

Our findings that longer stay at the ICU, sometimes considered as a proxy for severe disease^42^, was found to be a predictor for being physically inactive, at one year follow-up goes in line with existing literature^43,44^ as well as clinical reasoning. A past study found that prolonged hospital stay for COVID-19 predicted higher incidence of medical problems at one year follow-up^43^, although physical activity level was not an outcome in this study and the sample included all hospitalized individuals. There is suggestive evidence that more severely affected individuals tend to have longer ICU stays due to COVID-19^44^, although conclusive proof is lacking. Interestingly, females in the current study had significantly shorter stay at the ICU compared to males.

Diabetes mellitus and post-COVID syndrome are likely to have bidirectional relationship^45^. Diabetes could influence the course of COVID-19 by exacerbating tachycardia, microvascular dysfunction, and muscle fatigue^45–47^, which could affect the individual’s physical activity. Similarly, post-COVID-19 syndrome can add on to complications of diabetes such as tachycardia and fatigue^45^. In the current study, diabetes mellitus was a predictor for being physically inactive, as assessed using the SGPALS, but not using IPAQ-SF. Based on our results, it appears that people with diabetes mellitus and with a longer stay at ICU should be identified as a risk group for being physically inactive and be provided long-term follow-ups as well as enhanced support from healthcare providers to engage in physical activity. The chronic nature of diabetes mellitus can be speculated to impact long-term physical activity as assessed by SGPALS, but not short-term physical activity as assessed by IPAQ-SF.

The strength of this study is the well-defined cohort and the consecutive inclusion of survivors of severe COVID-19 during the pandemic’s first wave from Sweden’s second largest county in terms of population. In Sweden, no lockdown was implemented, due to which the assessments were not affected by the same. Our results enable the early identification of risk groups prone to physical inactivity after severe COVID-19, specifically individuals with longer ICU stays and diabetes. The results of the study can be generalized to one-year survivors of the first wave of COVID-19 in Western Sweden. A limitation of this study is that the sample size for the modelling is on the small side, and that we used self-reported questionnaires, where there is a risk for recall bias. This study does not consider factors that might influence physical inactivity after discharge from the ICU, as the aim of the study was to find the early predictors of physical inactivity. Slightly more than half of the potential participants (52.2% and 57.7%) responded to the questionnaires, which could have resulted in selection bias. However, we did not find significant differences between the responders and non-responders in terms of age, sex or length of stay at the ICU. Information on body mass index (BMI) at admission was missing for several participants in the medical records, so any association between BMI and physical inactivity at one year following severe COVID-19 could, unfortunately, not be established. The causal relationship between physical inactivity and the potential predictors could not be established in the current study as is the case in all observational studies. The proportion of individuals who developed ARDS in the current study is approximately 50–63%, which is lower than expected given that a global survey indicated around 75% incidence of ARDS in ICU admitted individuals for COVID-19^48^. We speculate that ARDS was underreported in the medical records in the participating ICUs due to the overwhelming state at the ICUs immediately following the pandemic. Furthermore, we do not have a control group and information regarding the pre-COVID-19 physical activity level, due to which it is not possible to conclude if physical inactivity at the time of follow-up was due to ICU admission or COVID-19 or both.

In conclusion, approximately a quarter of the participants were identified as being physically inactive one year following ICU care due to COVID-19. Longer stay in the ICU and presence of diabetes mellitus are associated with physical inactivity at one year following ICU admission for COVID-19.

## Electronic supplementary material

Below is the link to the electronic supplementary material.

Supplementary Material 1

## Data Availability

In response to a reasonable request, the dataset is available from the principal investigator, Carina U Persson (carina.persson@neuro.gu.se). Permission to use data can be obtained after an application to and approval by the Swedish Ethical Review Authority, according to Swedish regulations.
