# Supplementary material for: Physical activity level one year following admission to the intensive care unit for COVID-19
Source: Sci Rep. 2025 Apr 22;15:13916. doi: 10.1038/s41598-025-96775-0 (PMC12015298; doi:10.1038/s41598-025-96775-0)
Supplement: Supplementary file 1 — Supplementary Material 1 [file 41598_2025_96775_MOESM1_ESM.pdf]

Supplementary file

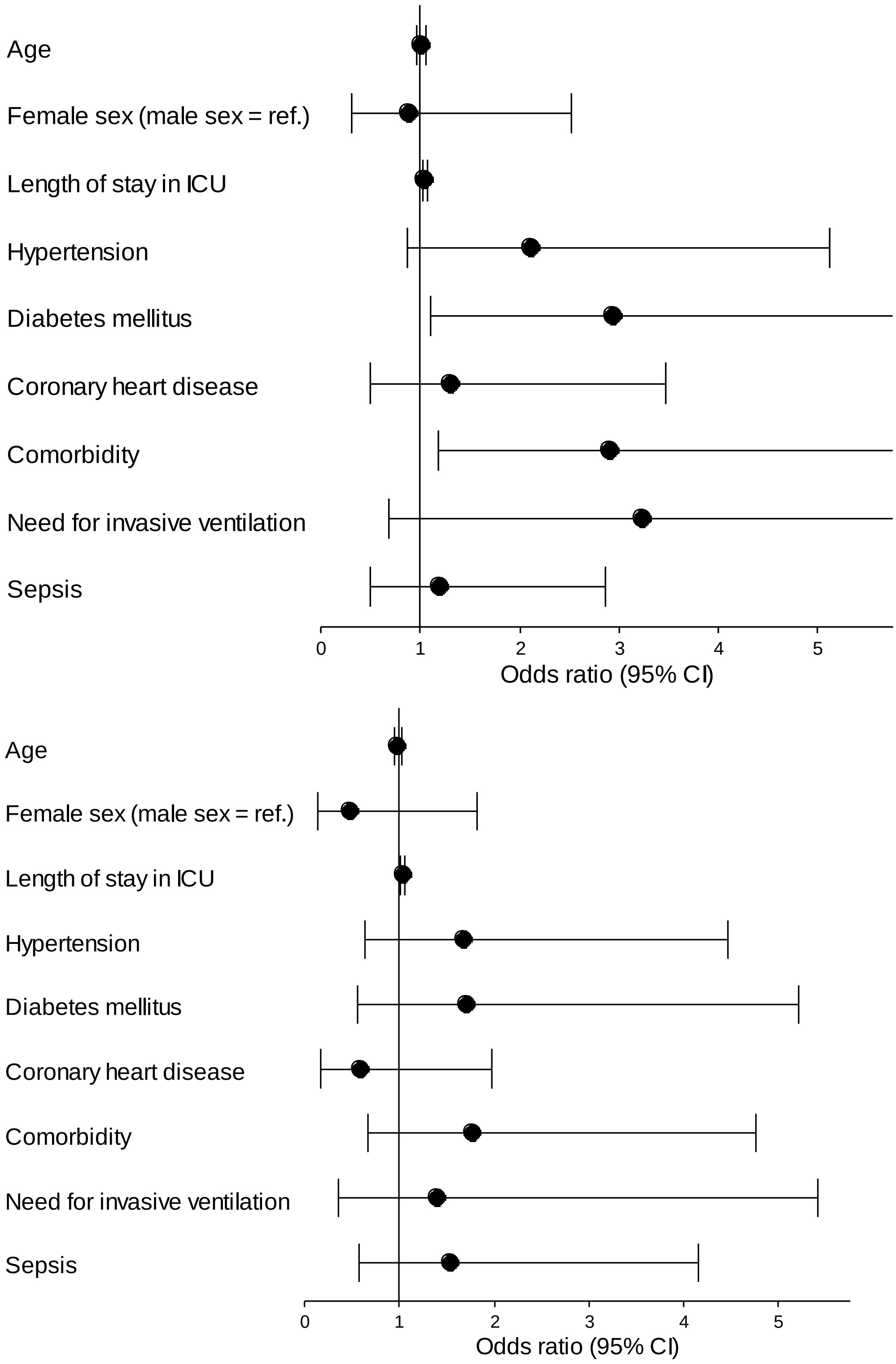

Figure 1. Forest plots based on the univariable regression analyses (also including comorbidity as one of the independent variables) with SGPALS (upper panel) and IPAQ-SF (lower panel) as the dependent variables showing the odds ratios and the 95% confidence intervals for the prediction of physical inactivity at one year following admission to the intensive care unit for COVID-19.

Supplementary file

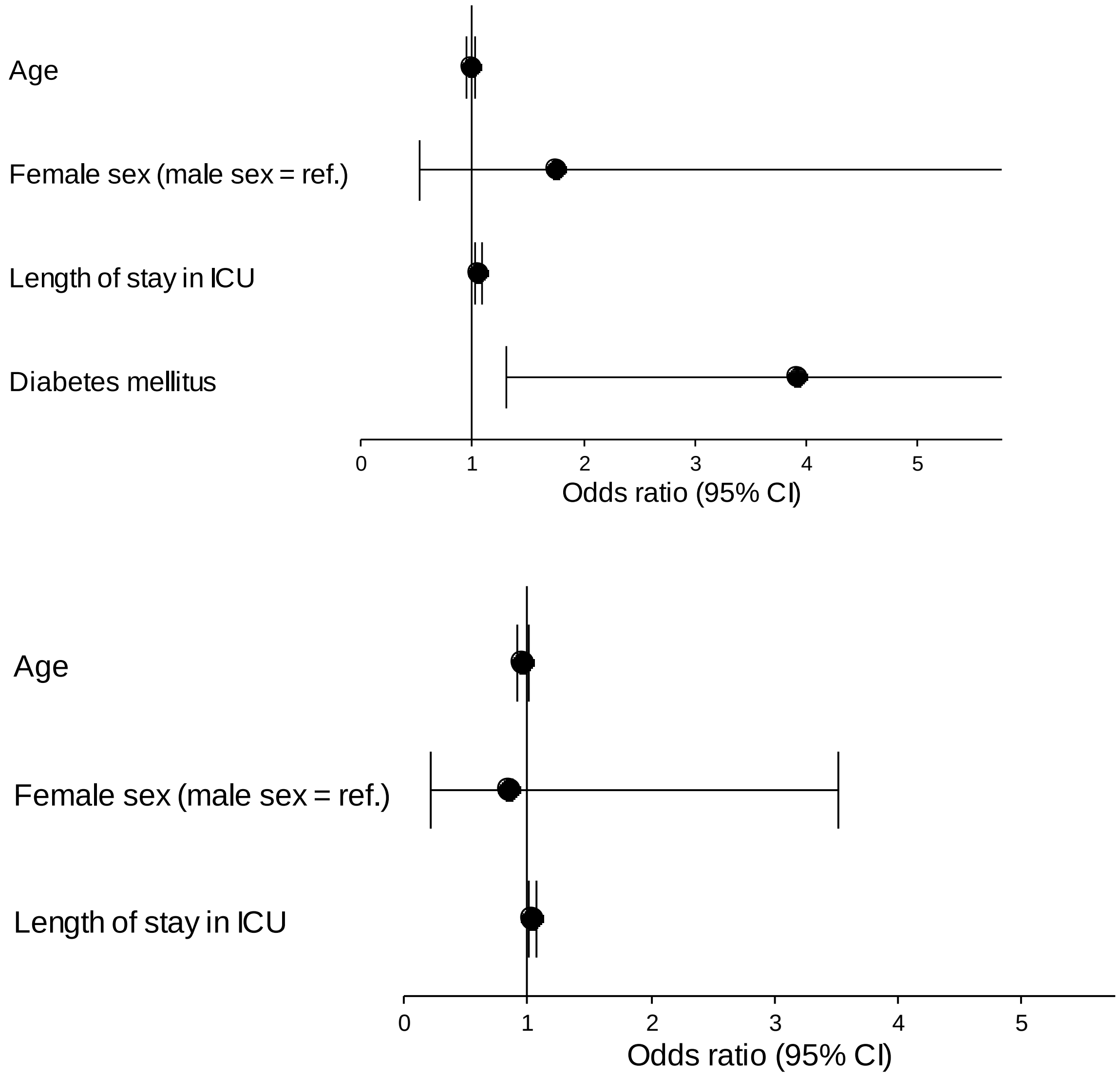

Figure 2. Forest plots based on the multivariable regression analyses showing the odds ratios and the 95% confidence intervals with SGPALS (upper panel) and IPAQ-SF (lower panel) as the dependent variables for the prediction of physical inactivity at one year following admission to the intensive care unit for COVID-19.
